# Does a 6-point scale approach to post-treatment 18F-FDG PET-CT allow to improve response assessment in head and neck squamous cell carcinoma? A multicenter study

**DOI:** 10.1186/s41824-020-00077-9

**Published:** 2020-05-26

**Authors:** P. Bonomo, A. Merlotti, S. Morbelli, V. Berti, C. Saieva, F. Bergesio, A. Bacigalupo, L. Belgioia, C. Franzese, E. Lopci, A. Casolo, E. D’Angelo, D. Alterio, L. Travaini, L. Berretta, V. Pirro, S. Ursino, D. Volterrani, M. Roncali, F. Vigo, S. Cicchetti, F. Scalone, G. Belli, S. Cauda, I. Desideri, E. Russi, L. Livi, A. Bianchi

**Affiliations:** 1 Radiation Oncology, Azienda Ospedaliero – Universitaria Careggi, University of Florence, Largo Brambilla 3,, 50134 Florence, Italy; 2 Radiation Oncology, Azienda Ospedaliera S. Croce e Carle, Cuneo, Italy; 3 Nuclear Medicine, IRCCS Ospedale Policlinico San Martino, Genoa, Italy; 4 Nuclear Medicine, Azienda Ospedaliero – Universitaria Careggi, University of Florence, Florence, Italy; 5 Cancer Risk Factors and Lifestyle Epidemiology Unit, Institute for Cancer Research, Prevention and Clinical Network (ISPRO), Florence, Italy; 6 Medical Physics, Azienda Ospedaliera S. Croce e Carle, Cuneo, Italy; 7 Radiation Oncology, IRCSS Ospedale Policlinico San Martino, Genoa, Italy; 8 Department of Radiotherapy and Radiosurgery, Humanitas Clinical and Research Hospital - IRCSS, Rozzano, Milan, Italy; 9 Nuclear Medicine, Humanitas Clinical and Research Hospital – IRCCS, Rozzano, Milan, Italy; 10 Nuclear Medicine, University Hospital of Modena, Modena, Italy; 11 Radiation Oncology, University Hospital of Modena, Modena, Italy; 12 Radiation Oncology, IEO European Institute of Oncology IRCCS, Milan, Italy; 13 Nuclear Medicine, IEO European Institute of Oncology IRCCS, Milan, Italy; 14 Radiation Oncology, Azienda Ospedaliera SS Antonio e Biagio e C. Arrigo, Alessandria, Italy; 15 Nuclear Medicine, Azienda Ospedaliera SS Antonio e Biagio e C. Arrigo, Alessandria, Italy; 16 Radiation Oncology, Azienda Ospedaliero-Universitaria Pisana, Pisa, Italy; 17 Nuclear Medicine, Azienda Ospedaliero-Universitaria Pisana, Pisa, Italy; 18 Nuclear Medicine, AUSL - IRCCS di Reggio Emilia, Reggio Emilia, Italy; 19 Radiation Oncology, AUSL - IRCCS di Reggio Emilia, Reggio Emilia, Italy; 20 Radiation Oncology, Tor Vergata University Hospital, Rome, Italy; 21 Nuclear Medicine, Tor Vergata University Hospital, Rome, Italy; 22 Radiation Oncology, Candiolo Cancer Institute, FPO- IRCCS, Candiolo, Turin, Italy; 23 Nuclear Medicine, Candiolo Cancer Institute, FPO- IRCCS, Candiolo, Turin, Italy; 24 Nuclear Medicine, Azienda Ospedaliera S. Croce e Carle, Cuneo, Italy

**Keywords:** Head and neck cancer, Radiotherapy, ^18^F-fluorodeoxyglucose (FDG) positron emission tomography/computed tomography, Diagnostic accuracy, Positive predictive value

## Abstract

**Purpose:**

Response assessment to definitive non-surgical treatment for head and neck squamous cell carcinoma (HNSCC) is centered on the role of ^18^F-fluorodeoxyglucose (FDG) positron emission tomography/computed tomography (PET-CT) 12 weeks after treatment. The 5-point Hopkins score is the only qualitative system available for standardized reporting, albeit limited by suboptimal positive predictive value (PPV). The aim of our study was to explore the feasibility and assess the diagnostic accuracy of an experimental 6-point scale (“Cuneo score”).

**Methods:**

We performed a retrospective, multicenter study on HNSCC patients who received a curatively-intended, radiation-based treatment. A centralized, independent qualitative evaluation of post-treatment FDG-PET/CT scans was undertaken by 3 experienced nuclear medicine physicians who were blinded to patients’ information, clinical data, and all other imaging examinations. Response to treatment was evaluated according to Hopkins, Cuneo, and Deauville criteria. The primary endpoint of the study was to evaluate the PPV of Cuneo score in assessing locoregional control (LRC). We also correlated semi-quantitative metabolic factors as included in PERCIST and EORTC criteria with disease outcome.

**Results:**

Out of a total sample of 350 patients from 11 centers, 119 subjects (oropharynx, 57.1%; HPV negative, 73.1%) had baseline and post-treatment FDG-PET/CT scans fully compliant with EANM 1.0 guidelines and were therefore included in our analysis. At a median follow-up of 42 months (range 5-98), the median locoregional control was 35 months (95% CI, 32-43), with a 74.5% 3-year rate. Cuneo score had the highest diagnostic accuracy (76.5%), with a positive predictive value for primary tumor (Tref), nodal disease (Nref), and composite TNref of 42.9%, 100%, and 50%, respectively. A Cuneo score of 5-6 (indicative of residual disease) was associated with poor overall survival at multivariate analysis (HR 6.0; 95% CI, 1.88-19.18; *p* = 0.002). In addition, nodal progressive disease according to PERCIST criteria was associated with worse LRC (OR for LR failure, 5.65; 95% CI, 1.26-25.46; *p* = 0.024) and overall survival (OR for death, 4.81; 1.07-21.53; *p* = 0.04).

**Conclusions:**

In the frame of a strictly blinded methodology for response assessment, the feasibility of Cuneo score was preliminarily validated. Prospective investigations are warranted to further evaluate its reproducibility and diagnostic accuracy.

## Introduction

Head and neck squamous cell carcinoma (HNSCC) is the sixth most common non-skin cancer worldwide (Rettig & D’Souza, 2015). In over 60% of cases, a non-metastatic locally advanced disease is found at diagnosis. Since about 20 years (Pignon et al., 2000; Pignon et al., 2009), concurrent chemo-radiotherapy (CRT) is the non-surgical mainstay of treatment for unresectable disease and organ preservation purpose. Historically, a 5-year survival rate of about 50% has been reported (Carvalho et al., 2005; Bathia & Burtness, 2015). Response evaluation to CRT is of critical importance for HNSCC management. In this respect, it is commonly recognized that morphologic imaging modalities may be suboptimal (Bhatnagar et al., 2013), particularly due to fibrosis, edema, and inflammatory changes mainly induced by radiation (RT). In analogy to non-small cell lung cancer (Iravani et al., 2019) and Hodgkin lymphoma (HL) (Cheson et al., 2014), metabolic information provides undisputed benefit to aid treatment response assessment in HNSCC. In particular, level one evidence (Mehanna et al., 2016) supports the notion that surveillance based on a negative ^18^F-fluorodeoxyglucose (FDG) positron emission tomography/computed tomography (FDG-PET/CT) acquired at a minimum of 12 weeks after CRT is non-inferior to an invasive strategy based on planned neck dissection in terms of long-term survival. The importance to apply standardized measures for FDG-PET/CT assessment has been highlighted by the widespread clinical use of Deauville criteria in HL management (Barrington & Kluge, 2017), whereby the metabolic pattern of interim response to chemotherapy (CHT) was demonstrated (Barrington et al., 2019) to be correlated with long-term outcome. In the setting of HNSCC, consistent qualitative evaluations in the post-treatment scenario are scarce. In this regard, the only recognized scoring system is represented by the “Hopkins criteria,” firstly introduced by Marcus et al. (Marcus et al., 2014). In a retrospective single-center study on 214 HNSCC patients treated at Johns Hopkins University in a time span of 13 years (2000-2013), the authors were able to show that a prespecified 5-point scale allowed to discriminate a complete versus an incomplete response to RT or CRT with a negative predictive value (NPV) and overall diagnostic accuracy of 91.1% and 86.9%, respectively. In a prospective, multicenter study (Van Den Wyngaert et al., 2017) focused on the standardized implementation and reporting of FDG-PET/CT acquired at 12 weeks after CRT, the use of Hopkins criteria yielded a NPV of 92.1%. Overall, the excellent diagnostic performance associated with a negative FDG-PET/CT scan is however counterbalanced by its suboptimal positive predictive value (PPV), an unresolved issue in head and neck oncology. To compensate for this known limitation, integrating metabolic information with clinical data and morphologic imaging is essential for proper response assessment and surveillance. In a meta-analysis (Gupta et al., 2011) on 2335 patients from 51 studies, a mean pooled PPV of 58.6% was obtained. A more recent meta-analysis (Helsen et al., 2018) from 20 studies (1293 subjects) yielded the same PPV (58%). In both Johns Hopkins retrospective experience (Marcus et al., 2014) and ECLYPS study (Van Den Wyngaert et al., 2017), a PPV of 71.1% and 62.5% was reported, respectively. In this perspective, the discriminative power of Hopkins score may be inherently limited. We hypothesized that the potential limitation of a 5-point scoring system such as the Hopkins criteria may be overcome by a more sensitive 6-point scale discriminating patients with complete, incomplete or equivocal response to treatment, identifying a threshold score of response for each of the 3 patterns. Ideally, it would be noteworthy to apply standardized diagnostic criteria able to minimize false negative but also false-positive results: a high PPV may allow to better address the common development of inflammatory reactions after RT and the slow nodal disease regression of HPV positive disease (Huang et al., 2013), factors known to impair the FDG-PET/CT evaluation 12 weeks after treatment in both the PET-NECK (Mehanna et al., 2016) and ECLYPS (Van Den Wyngaert et al., 2017) trials. The aim of our study was to test the reproducibility and evaluate the diagnostic accuracy of an experimental 6-point scale in assessing the response to curatively—intended treatment for HNSCC. In addition, we sought to investigate whether baseline semi-quantitative metabolic factors or their change after treatment (“delta”) as included in PERCIST (JH et al., 2016) and EORTC (Young et al., 1999) criteria correlated with disease outcome.

## Methods

### Design and setting of the study

We performed an observational, retrospective, multi-center study within the Italian Association of Radiotherapy and Clinical Oncology (AIRO). In order to overcome the potential limitations of retrospective assessments in the field of functional imaging, a rigorous centralized review of FDG-PET/CT scans was mandated per protocol and performed by 3 independent nuclear medicine physicians (AB, SM, VB). The study was approved by the local ethics committees of each participating center. Participating Nuclear Medicine centers needed to have clinical trial qualification released from the “Federazione Italiana Linfomi” (FIL) core lab (Chauvie et al., 2016).

### Patients’ characteristics

Consecutive patients treated between 1/1/10 and 31/12/15 could be enrolled in our study. Performance status (PS) according to Eastern Cooperative Oncology Group (ECOG), smoking history and comorbidity profile according to Charlson comorbidity index (CCI) (Charlson et al., 1987) were recorded at HNSCC diagnosis. Patients with non-metastatic, histologically proven squamous cell carcinoma of the oropharynx, hypopharynx, larynx and nasopharynx, or undifferentiated nasopharyngeal carcinoma candidate to non-surgical treatment with curative intent could be included. Pending inclusion in the study, a baseline and post-treatment FDG-PET/CT examinations had to be available for each subject. By definition, the baseline scan had to be executed not before 8 weeks from the start of treatment (first day of RT or first cycle of induction CHT, if performed). The post-treatment scan had to be executed not before 10 weeks and no later than 6 months after the end of treatment (last RT fraction)**.** Clinical stage was defined based on TNM/AJCC 7th edition. Human papilloma virus (HPV) status was not routinely available in all centers in the considered timeframe. HPV positivity was defined by a 70% diffuse nuclear staining detected by p16 immunohistochemistry, with additional HPV-DNA in situ hybridazation as confirmatory test in selected cases. A curatively intended treatment was defined as one of the following: RT alone; cisplatin-based concurrent CRT; cetuximab-based concurrent CRT; induction CHT followed by RT alone; induction CHT followed by concurrent CRT. Prior RT to the head and neck region and gross total excision of both primary and/or nodal disease before index treatment were not allowed. No upper age limit was defined.

### FDG-PET/CT analysis

Only FDG-PET/CT scans performed according to EANM 1.0 procedure guidelines (Boellard et al., 2010) were evaluated. No contrast media for the CT component of FDG PET/CT examinations were used. Moreover, a strict criterion for uptake time was applied. In fact, only patients whose both baseline and post-treatment scans were acquired within the range of 60 + 10 min after FDG injection were ultimately included in our study (supplementary material).

An independent evaluation of all FDG-PET/CT images was undertaken by 3 experienced nuclear medicine physicians who were blinded to patients’ information, clinical data, and other imaging examinations. Anthropometric variables (weight, height, and sex) and selected technical parameters (administered activity of FDG per kilogram, plasma glucose level at time of scan, uptake time) were automatically provided for each case. A qualitative assessment of all post-treatment scans was performed in accordance with 2 standardized reporting systems, Hopkins and Deauville criteria. In addition, an experimental 6-point score (“Cuneo score” was tested (Table 1)). Deauville criteria were experimentally applied to HNSCC. The 3 scores were determined by each reviewer for the primary tumor (Tref) and nodal disease (Nref), respectively. A composite TNref score was also calculated, corresponding to the highest score reported. By definition, the diagnostic accuracy of the 3 qualitative scores was assessed on all those cases where an agreement of at least 2 of the 3 reviewers was found. To this end, a secondary revision of selected cases without initial agreement of at least 2 reviewers was allowed prior to the final analysis to rule out major discrepancies. Response according to PERCIST and EORTC criteria was also computed (supplementary material).

**Table 1:**
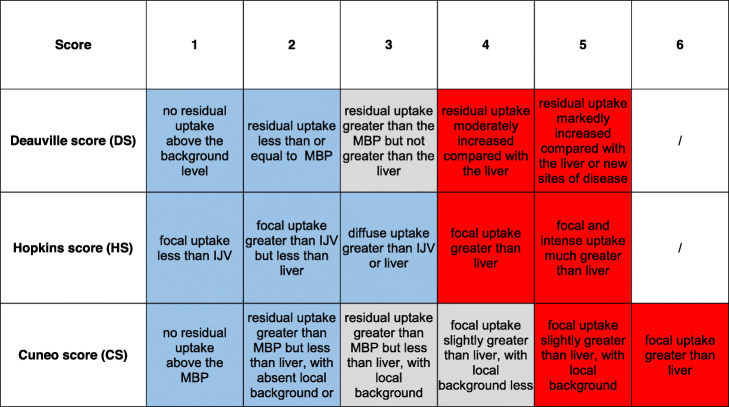
Standardized reporting criteria used to assess FDG-PET/CT scans (Deauville, Hopkins, and Cuneo scores)

Light blue, red, and light gray boxes corresponding to scores indicative of absence of disease, presence of disease or equivocal finding, respectively

### Statistical analysis

Inter-observer agreement was measured with the Krippendorff’s alpha coefficient at 3-time points: after a blinded review of a “training set” of 15 patients (phase 1), after a second review of the “training set” (phase 2), and after the revision of the whole cohort enrolled (phase 3). After feedback from phase 1, a meeting was held to discuss interpretation, and a detailed set of instructions for the review procedure was agreed and acted upon.

Response to treatment was assessed by local investigators in accordance with the RECIST 1.1 criteria (Therasse et al., 2000). Progression-free survival (PFS) was defined as the time from the last day of treatment to the date of the first of the following events: the first day when criteria for progressive disease (PD) are met; salvage surgery or elective neck dissection after 15 weeks from the last day of RT performed on the clinical or radiological evidence of progression; death for any cause. The type of first PFS event was selected among the following: local failure (i.e, primary tumor); regional failure (i.e, lymph nodes); simultaneous local and nodal failure; distant failure; second primary tumor. Loco-regional control (LRC) was defined as the time from the last day of treatment to the date of the first loco-regional event. Overall survival (OS) was defined as the time from the date of HNSCC diagnosis to death from any cause or last follow-up. LRC, PFS, and OS were estimated by the Kaplan-Meyer method. In order to evaluate whether specific patient, disease, and treatment features or FDG-PET/CT response pattern had a potential prognostic impact on outcome, Cox regression analysis was performed, by calculation of hazard ratios (HR) and corresponding 95% confidence intervals (CI 95%). A multivariate analysis was performed when multiple risk factors with a *p* value < 0.05 were identified in the univariate analysis. Median LRC, PFS, and OS and their estimates at 36 months were calculated with corresponding 95% CI. For each standardized qualitative score (Deauville, Hopkins, and Cuneo) applied to post-treatment scans, we calculated the diagnostic accuracy for Tref, Nref, and composite TNref. It was expressed in terms of sensitivity (SE), specificity (SP), PPV, NPV, and overall accuracy. The semi-quantitative metabolic parameters were expressed as delta value, defined as the difference of standardized uptake value corrected for body weight (SUV/bw) and for lean body mass (SUL) values between the baseline and post-treatment scans, for both Tref and Nref. Descriptive values are presented as mean (⌖SD), median, range, and tertiles. Mann-Whitney test was used to evaluate the difference on selected mean delta values by specific outcomes (LRC, PFS, and OS). Categorical variables were also calculated according to median and tertiles values, respectively. The association between categorical delta values and the outcomes (LRC, PFS, and OS) was evaluated by simple cross-tables and appropriate chi-square test. Logistic models were also performed to confirm the possible association by odd ratios (OR) and 95% CI calculation. Chi-square test and logistic models were also used to evaluate the association between PERCIST (JH et al., 2016) and EORTC (Young et al., 1999) criteria and the outcomes (LRC, PFS, and OS). Differences were considered statistically significant at the level of *p* < 0.05. Statistical analyses were performed using the IBM SPSS Statistics software (Statistical Package for Social Science, version 22).

The primary endpoint of the study was to evaluate the PPV of Cuneo score in assessing LRC after treatment. The secondary endpoints were to evaluate the overall diagnostic accuracy of Cuneo, Deauville and Hopkins criteria in response assessment; the rate of interobserver agreement of Cuneo score assessed with Krippendorff’s alpha; the correlation of FDG-PET/CT semiquantitative body-weighted and lean-body mass parameters at baseline scans and their delta (percentage of change) with LRC, PFS, and OS; and the correlation of response assessment according to EORTC and PERCIST criteria with LRC, PFS, and OS. Considering a PPV of 71.1% with post-treatment FDG-PET/CT based on the work of Marcus et al. (Marcus et al., 2014), applying the experimental 6-point Cuneo score was hypothesized to yield a 20% increase. It was therefore assumed the null hypothesis (H0) that the PPV is 71.1% versus the alternative hypothesis (Ha) that the PPV is 85.3%. With a significance level *α* = 0.05 and a power of 0.90 when the PPV is 85.3%, the required sample size with this design was 81 patients.

## Results

### Patients’ characteristics and treatment outcome

**Fig. 1:**
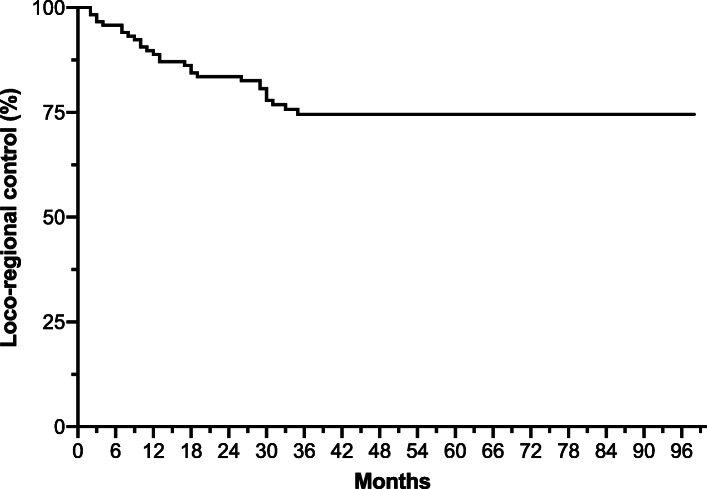
Loco-regional control (Kaplan-Meyer method)

Between Jan. 2010 and Dec. 2015, a total of 350 patients from 11 centers were included in our study. In view of the fact that the requested acquisition time of all PET scans should be 60 + 10 min. after injection, our final cohort consisted of 119 subjects. Patients’ characteristics are shown in Table 2. At diagnosis, the median age was 60 years (range 19-84), with most patients in good general conditions (ECOG PS of 0-1 in 96.6% of cases) and a baseline median age-adjusted CCI of 4. The most common primary site was oropharynx (57.1%): overall, most patients in our study had a locally advanced disease (91.5% with stage III-IV) and HPV negative status (73.1%). Primary treatment was heterogeneous, with concurrent CRT as prevalent modality (54.6%). No patients underwent a planned neck dissection. The mean time intervals from baseline PET execution to start of treatment and from its end to final PET execution were 3.1 and 16.6 weeks, respectively. At a median follow-up of 42 months (range 5-98), the median LRC, PFS, and OS were 35 (95% CI, 32-43), 33 (95% CI, 30-40), and 45.5 (95% CI, 39-51) months, respectively. The 3-year rates of LRC, PFS, and OS were 74.5%, 61.3%, and 82.2%, respectively (Fig. 1 and supplementary figures 1-2). Overall, 28/119 patients (23.5%) were censored for loco-regional recurrence.

**Table 2:** Patients’ disease and treatment characteristics

Characteristic	No. of patients (%) ***n*** = 119
**Median age**, years (range)	60 (19-84)
**Sex**	
Male	88 (73.9)
Female	31 (26.1)
**ECOG performance status**	
0	80 (67.2)
1	35 (29.4)
2	4 (3.4)
**Charlson comorbidity index (age-adjusted)**	
< 4	53 (44.5)
4-7	61 (51.3)
≥ 8	5 (4.2)
**Smoking history (pack/years)**	
0	19 (21.2)
< 10	15 (16.6)
10-20	11 (12.2)
> 20	45 (50.0)
**Primary tumor**	
Oropharynx	68 (57.1)
Larynx	19 (15.8)
Hypopharynx	10 (8.5)
Nasopharynx	22 (18.6)
**HPV status** (oropharynx only)	
Positive	32 (47.0)
Negative	22 (32.4)
Unknown	14 (20.6)
**T stage at diagnosis (VIIth ed)**	
1	11 (9.3)
2	36 (30.2)
3	31 (26.0)
4a/4b	41 (34.5)
**N stage at diagnosis (VIIth ed)**	
0	26 (21.9)
1	19 (16.0)
2a/2b/2c	73 (61.4)
3	1 (0.7)
**AJCC stage at diagnosis (VIIth ed)**	
I-II	10 (8.4)
III	27 (22.7)
IVA	76 (63.9)
IVB	6 (5.0)
**Primary treatment modality**	
RT	14 (11.8)
Induction CT + RT	2 (1.7)
Induction CT + RCT	23 (19.3)
Induction CT + BRT	2 (1.7)
CRT	65 (54.6)
BRT	13 (10.9)

### FDG-PET/CT training phase

**Fig. 2:**
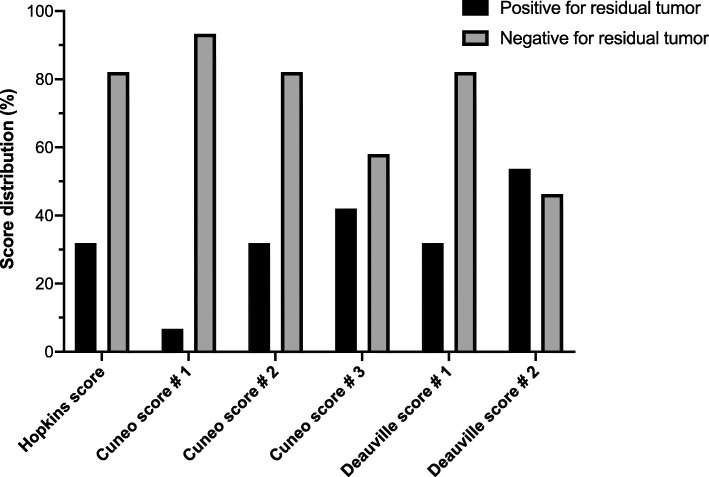
Score distribution of FDG-PET/CT post-treatment scans according to used standardized reporting criteria (Deauville, Hopkins and Cuneo scores). Post-treatment scans classified as positive or negative for residual tumor (black and grey bars, respectively) based on composite TNref score

The application of Hopkins, Cuneo, and Deauville scores for post-treatment response assessment yielded different score distributions, as shown in Fig. 2. Regarding the interobserver agreement, the Krippendorff’s alpha values for Tref and Nref evaluated at the 3-time points of assessment are reported in Table 3.

**Table 3:** Krippendorff’s alpha values for inter-observer agreement throughout 3 rounds of revision of post-treatment FDG-PET/CT scans for Tref (defined as the area with highest FDG uptake within the residual primary tumor) and Nref (defined as the residual lymph node with highest FDG uptake)

	TRef			NRef		
	Deauville score	Hopkins score	Cuneo score	Deauville score	Hopkins score	Cuneo score
Phase 1	0.29	0.09	0.31	0.02	0.07	0.15
Phase 2	0.49	0.37	0.36	0.47	0.41	0.58
Phase 3	0.48	0.45	0.34	0.40	0.38	0.36

### Accuracy of FDG-PET/CT-based qualitative scores

In light of the reported good interobserver agreement, we then assessed as primary endpoint the diagnostic accuracy of the 3 qualitative reporting scales to rule out loco-regional failure at post-treatment scan, primarily in terms of PPV (Table 4, supplementary table 1). The equivocal scores (3 and 4 according to Cuneo score and 3 according to Deauville score) were clustered at time of analysis with definite positive and negative findings in order to identify the best diagnostic threshold. Overall, Cuneo score no. 1 (with scores 3 and 4 clustered with 1 + 2, indicative of absence of disease) yielded the best PPV for all categories (Tref, Nref, and TNref of 42.9%, 100%, and 50%, respectively). In addition, it also had the highest TNref overall accuracy (76.5%), followed by Cuneo score no. 2 (score 3 clustered with 1 + 2, score 4 clustered with 5 + 6, indicative of persistent disease), Hopkins score, Deauville score no. 1 (score 3 clustered with 1 + 2, indicative of absence of disease), Cuneo score no. 3 (scores 3 and 4 clustered with 5 + 6, indicative of residual disease), and Deauville score no. 2 (score 3 clustered with 4 + 5, indicative of residual disease) with 68.1%, 68.1%, 68.1%, 61.3%, and 54.6%, respectively. Pictorial examples of Tref and Nref scoring with the 3 scales are shown in supplementary figures 3 and 4, respectively.

**Table 4:** Overall diagnostic accuracy using standardized reporting criteria (Deauville, Hopkins, and Cuneo scores)

**Hopkins score (HS)**	**Tref**	**Nref**	**TNref**
1, 2, 3: absent disease			
4, 5: persistent disease			
**Characteristic**			
Sensitivity	39.3	10.7	50.0
Specificity	75.8	96.7	73.6
NPV	80.2	77.9	82.7
PPV	33.3	50.0	36.8
Overall accuracy	67.2	76.5	68.1
**Cuneo score (CS # 1)**	**Tref**	**Nref**	**TNref**
1, 2, 3, 4: absent disease			
5, 6: persistent disease			
**Characteristic**			
Sensitivity	10.7	35.7	14.3
Specificity	95.6	100	95.6
NPV	77.7	77.1	78.4
PPV	42.9	100	50.0
Overall accuracy	75.6	77.5	76.5
**Cuneo score (CS # 2)**	**Tref**	**Nref**	**TNref**
1, 2, 3: absent disease			
4, 5, 6: persistent disease			
**Characteristic**			
Sensitivity	39.3	10.7	50
Specificity	75.8	96.7	73.6
NPV	80.2	77.9	82.7
PPV	33.3	50	36.8
Overall accuracy	67.2	76.5	68.1
**Cuneo score (CS # 3)**	**Tref**	**Nref**	**TNref**
1, 2: absent disease			
3, 4, 5, 6: persistent disease			
**Characteristic**			
Sensitivity	53.6	17.9	57.1
Specificity	64.8	94.5	62.6
NPV	81.9	78.9	82.6
PPV	31.9	50	32
Overall accuracy	62.2	76.5	61.3
**Deauville score (DS # 1)**	**Tref**	**Nref**	**TNref**
1, 2, 3: absent disease			
4, 5: persistent disease			
**Characteristic**			
Sensitivity	39.3	10.7	50.0
Specificity	75.8	96.7	73.6
NPV	80.2	77.9	82.7
PPV	33.3	50.0	36.8
Overall accuracy	67.2	76.5	68.1
**Deauville score (DS # 2)**	**Tref**	**Nref**	**TNref**
1, 2: absent disease			
3, 4, 5: persistent disease			
**Characteristic**			
Sensitivity	64.3	17.9	67.9
Specificity	54.9	85.7	50.5
NPV	83.3	77.2	83.6
PPV	30.5	27.8	29.7
Overall accuracy	57.1	69.7	54.6

*NPV* negative predictive value, *PPV* positive predictive value

### Prognostic impact of FDG-PET/CT semi-quantitative parameters

In order to evaluate whether any change of semi-quantitative parameters over time could be of prognostic impact, delta values for Tref and Nref between baseline and post-treatment scans were correlated with LRC, PFS, and OS. For this purpose, 109 patients were analyzed, since 10 subjects were excluded due to the fact that the baseline FDG-PET/CT was performed during or at the completion of induction CHT. Patients with a SUV_max_ reduction for Nref in the second tertile had a lower risk of death compared to first and third tertiles (*p* = 0.046; OR for death, 0.27; 95% CI, 0.09-0.85, *p* = 0.025). All other variables (either as continuous or categorical) were not significantly associated with efficacy outcomes. When standardized semi-quantitative response assessments were applied, no correlation could be found between EORTC criteria and LRC, PFS, and OS, whereas nodal PD according to PERCIST criteria was associated with a higher risk of loco-regional failure (*p* = 0.026; OR for LR failure, 5.65; 95% CI, 1.26-25.46, *p* = 0.024) and death (*p* = 0.04; OR for death, 4.81; 1.07-21.53, *p* = 0.04).

At Cox regression univariate analysis, the use of induction (*p* = 0.035) and concurrent CHT (*p* = 0.0001) correlated with better OS, whereas a higher risk of death was found for patients with baseline PS 2 (*p* = 0.001). In addition, a Cuneo score of 5-6 was also indicative of poor prognosis (*p* = 0.0001), which retained statistical significance at multivariate analysis (HR 6.0; 95% CI, 1.88-19.18; *p* = 0.002). No correlation was observed in respect with CCI (< or ≥ 4), stage (IV vs others) and HPV status.

## Discussion

Supported by high level of evidence (Mehanna et al., 2016), the use of FDG-PET/CT after definitive CRT is recommended for HNSCC; however, the lack of a validated interpretation system prevents from cross-comparisons among studies and accurate prognostication in clinical practice. In the present multicenter study, we preliminarily validated the use of Cuneo score, a 6-point qualitative scale by assessing its feasibility and inter-reader agreement and by demonstrating an improvement in PPV after CRT with respect to other score-based approaches previously proposed in this clinical setting. The clinical relevance of applying standardized qualitative criteria in treatment response assessment with FDG-PET/CT is epitomized by the widespread reproducibility and prognostic validity of Deauville score in HL (Kobe et al., 2018). In comparison to it, only limited data are thus far available for solid malignancies (Helsen et al., 2018; Scarsbrook et al., 2017; Huang et al., 2019). In this context, the Hopkins criteria represent the only proposed scoring system available for qualitative evaluation of post-treatment FDG-PET/CT in HNSCC. This 5-point scale is essentially based on the adoption of internal jugular vein (IJV) activity as blood-pool background reference. A well conducted prospective study (Van Den Wyngaert et al., 2017) showed that the application of Hopkins criteria allowed to obtain a lower rate of equivocal findings in comparison with non-standardized local read (1.6% vs 10.4%, *p* = .003), whereas the overall diagnostic accuracy was not improved (AUC of 0.78 and 0.73, respectively, *p* = .336). The available data (Marcus et al., 2014; Van Den Wyngaert et al., 2017) suggest that this scale may not be the best solution to address the inherently suboptimal PPV of FDG-PET/CT assessment in HNSCC. In this perspective, the rationale behind the design of the 6-tiered Cuneo score lied in replacing the activity of IJV as background reference with those of mediastinal blood pool (MBP) and liver and in comparing the FDG-avid spot with the local background. In case of residual uptake above the MBP, we envisaged to correlate the relationship between the focal uptake and local background with the liver activity, in order to take into account the known “contrast illusion effect” which may be particularly relevant in head and neck anatomy. Overall, we sought to design a scale with a more gradual shift in scoring based on the local background activity, aiming for a better clusterization of false positives compared with Hopkins criteria. Following a standardized concordance methodology widely adopted in HL scenario (Biggi et al., 2013), a good inter-reader agreement was achieved in our work. Despite its potentially high degree of complexity, the Cuneo score was prospectively shown to be feasible with adequate reproducibility. In addition, no difference was observed in terms of inter-reader agreement comparing Cuneo score with Hopkins and Deauville criteria. While Hopkins criteria were already externally validated in a single-center retrospective experience (Kendi et al., 2017) and ECLYPS study (Van Den Wyngaert et al., 2017) with excellent concordance, the experimental application of Deauville score to HNSCC response assessment is a peculiar finding of our work. Clearly, prospective studies are required to further confirm the reliability of Cuneo score for standardized reporting. As already mentioned, when the Cuneo score threshold differentiating absence and residual disease was set between scores 4 and 5 (CS no. 1), the highest PPV rates were obtained. In particular, achieving 100% nodal PPV is of extreme interest; in our opinion, this finding may lend support to the beneficial adoption of a 6-point scale essentially centered on the local background activity to rule out false-positive interpretations of irradiated lymph nodes, a critical issue for HNSCC. From a clinical perspective, the ability to discriminate with excellent accuracy the presence of residual nodal disease may allow the early implementation of salvage surgery, whereas the recognition of a false positive finding with high reliability may spare unnecessary morbidity avoiding a neck dissection. In contrast, the suboptimal PPV rate (42.9%) reported for primary tumor assessment underlines the importance of integrating two or more imaging modalities and clinical feedback for this specific purpose (Jentsch et al., 2015). Regarding NPV, the results obtained with Hopkins, Deauville, and Cuneo scores were similar, in the range of 80%, thus less than usually expected in this context. Since we did not limit our post-treatment evaluation to a 6-month timepoint, taking also into account that the mean time to loco-regional failure in our cohort was of 16 months and that 53% (15/28) of loco-regional recurrences occurred after 1 year from the end of RT, we think that the less than excellent NPV results reflect a time-dependent loss of accuracy to detect late relapses, a finding also shown in the ECLYPS study (Van Den Wyngaert et al., 2017). On top of excluding almost two-thirds of potential candidate patients from our retrospective analysis, our attempt to follow a rigorous centralized assessment implied that the 3 reviewers were blinded to all clinical data and could not examine computed tomography or magnetic resonance of any individual case, whenever performed. On the one hand aiming to increase the reviewers’ objectivity, on the other, we think that this was the main reason why all our qualitative interpretations underperformed in terms of PPV, particularly for Tref response assessment, falling short of what initially hypothesized. In other words, the strictly blinded review we performed could have contributed to skew our results towards worse overall accuracy, compared with what may be achieved in clinical practice. Additional limitations have to be acknowledged in the interpretation of our results. First, in spite of the outlined inclusion criteria, the retrospective nature of our study and the inherent clinical heterogeneity cannot be overlooked. Second, the HPV positive subgroup was much less represented that in the original paper by Marcus et al. (Marcus et al., 2014) (32 vs 123 patients, 26.9% vs 57.5% of the whole sample, respectively), thus restraining us from drawing correlations between the diagnostic performance of Cuneo score and the false positives related to the slow nodal clearance of HPV positive HNSCC. Third, the relatively low number of our sample and of loco-regional recurrences may limit the strength of our findings. Finally, no attempt was performed to correlate the post-treatment qualitative scores with nodal morphologic features (Wray et al., 2016) or radiation planning dosimetry (Morgan et al., 2019). When analyzing the semi-quantitative FDG-PET/CT variables and their delta values between pre- and post-treatment scans, no definitive conclusions can be drawn, except for the unfavorable prognostic impact of nodal progression according to PERCIST criteria. Notably, the significant correlation found between a Cuneo score indicative of persistent disease (5 and 6 according to Cuneo score no. 1) and poor OS reinforces the assumption that the application of standardized qualitative criteria may be well suited for clinical prognostication. Although our data need to be interpreted with caution in light of the peculiar methodology we followed, the results obtained by applying the Cuneo score after CRT in HNSCC are promising and deserve to be further investigated.

In conclusion, a standardized qualitative interpretation of FDG-PET/CT-based post-treatment assessment in HNSCC is still largely underrepresented in clinical practice. A 6-point scale such as the Cuneo score is feasible and may allow for better discrimination in respect to PPV, compared with the Hopkins criteria. To further elucidate its reproducibility and overall diagnostic accuracy, prospective studies are warranted.

## Supplementary information

**Additional file 1: figure 1.** progression-free survival (Kaplan Meyer method)

**Additional file 2: figure 2.** overall survival (Kaplan Meyer method)

**Additional file 3: figure 3.**: fused FDG-PET/CT images of a 67-year old male with a T4aN3 oropharyngeal cancer: Tref assessment negative for residual tumor according to all scales

**Additional file 4: figure 4.** fused FDG-PET/CT images of a 69-year old male with a T4aN2b laryngeal cancer: Nref assessment negative for residual tumor according to all scales

**Additional file 5: table 1.** positive predictive value and corresponding 95% CI of composite TNref for specific scores. PPV: positive predictive value

**Additional file 6: material.** FDG-PET/CT analysis

## Data Availability

Not applicable

## Abbreviations

AIRO: Italian Association of Radiotherapy and Clinical Oncology
AUC: Area under the curve
CCI: Charlson comorbidity index
CHT: Chemotherapy
CI: Confidence intervals
CRT: Chemo-radiotherapy
EANM: European Association of Nuclear Medicine
ECOG: Eastern Cooperative Oncology Group
FDG: ^18^F-fluorodeoxyglucose
FDG-PET/CT: ^18^F-fluorodeoxyglucose-positron emission tomography/computed tomography
FIL: Federazione Italiana Linfomi
HL: Hodgkin lymphoma
HNSCC: Head and neck squamous cell carcinoma
HPV: Human papilloma virus
HR: Hazard ratio
IJV: Internal jugular vein
LRC: Loco-regional control
MBP: Mediastinal blood pool
NPV: Negative predictive value
OR: Odd ratio
OS: Ooverall survival
PS: Performance status
PPV: Positive predictive value
PFS: Progression-free survival
PD: Progressive disease
RT: Radiation
SE: Sensitivity
SP: Specificity
