# Supplementary figures and images for: Does a 6-point scale approach to post-treatment 18F-FDG PET-CT allow to improve response assessment in head and neck squamous cell carcinoma? A multicenter study
Source: Eur J Hybrid Imaging. 2020 May 26;4:8. doi: 10.1186/s41824-020-00077-9 (PMC8218061; doi:10.1186/s41824-020-00077-9)

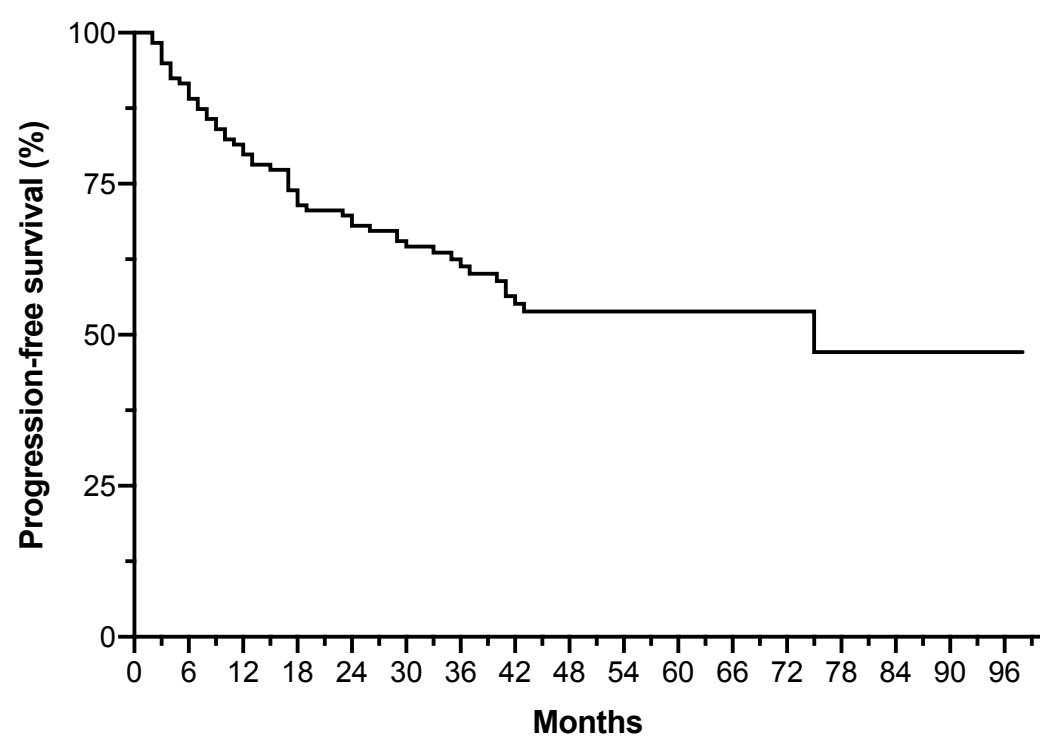

Supplement: Supplementary file 1 — Additional file 1: figure 1. progression-free survival (Kaplan Meyer method) [file 41824_2020_77_MOESM1_ESM.pdf]

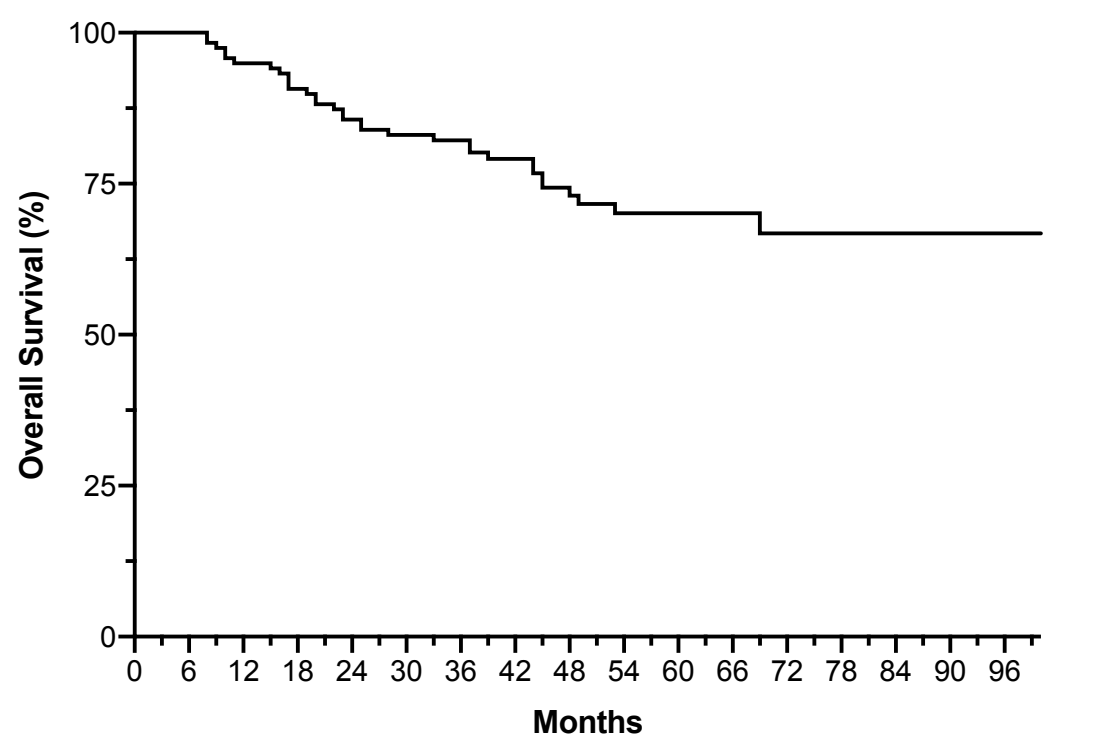

Supplement: Supplementary file 2 — Additional file 2: figure 2. overall survival (Kaplan Meyer method) [file 41824_2020_77_MOESM2_ESM.pdf]
