# Supplementary material for: Does a 6-point scale approach to post-treatment 18F-FDG PET-CT allow to improve response assessment in head and neck squamous cell carcinoma? A multicenter study
Source: Eur J Hybrid Imaging. 2020 May 26;4:8. doi: 10.1186/s41824-020-00077-9 (PMC8218061; doi:10.1186/s41824-020-00077-9)

Deauville	Hopkins	Cuneo
3 (negative)	2 (negative)	3 (negative)

Baseline

Post-treatment

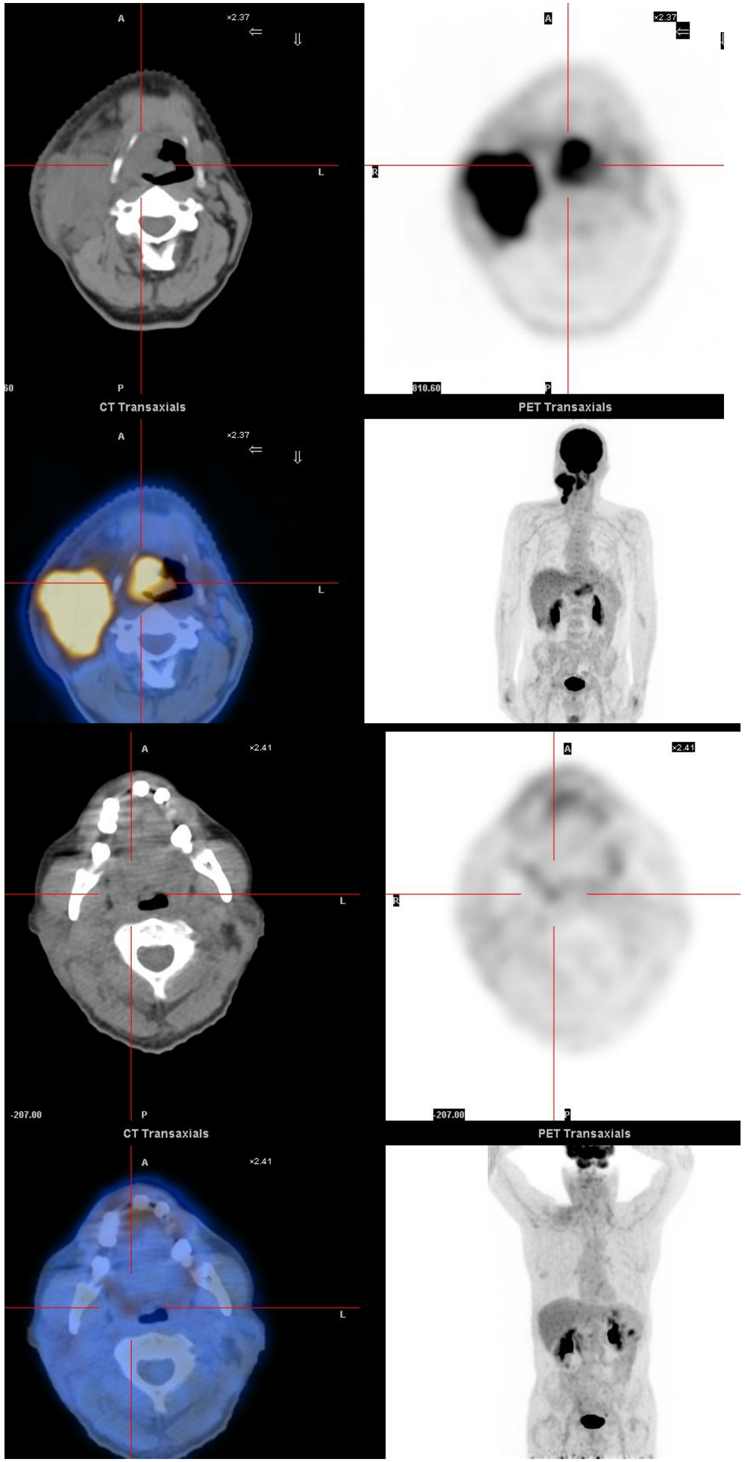

Post-treatment

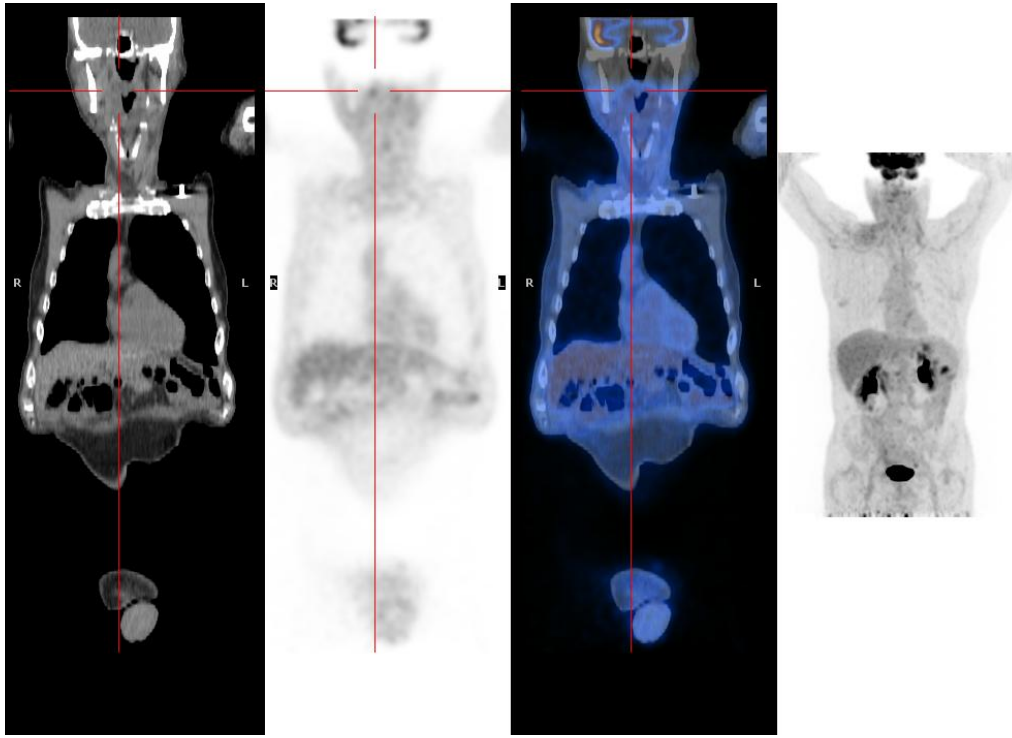

Supplement: Supplementary file 3 — Additional file 3: figure 3.: fused FDG-PET/CT images of a 67-year old male with a T4aN3 oropharyngeal cancer: Tref assessment negative for residual tumor according to all scales [file 41824_2020_77_MOESM3_ESM.pdf]
