# Supplementary material for: Does a 6-point scale approach to post-treatment 18F-FDG PET-CT allow to improve response assessment in head and neck squamous cell carcinoma? A multicenter study
Source: Eur J Hybrid Imaging. 2020 May 26;4:8. doi: 10.1186/s41824-020-00077-9 (PMC8218061; doi:10.1186/s41824-020-00077-9)

Baseline

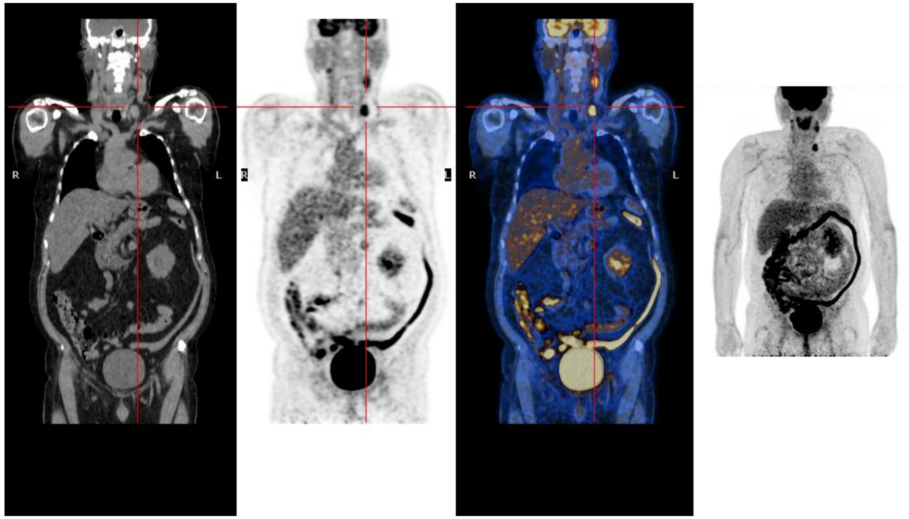

Deauville	Hopkins	Cuneo
3 (negative)	2 (negative)	2 (negative)

Post-treatment

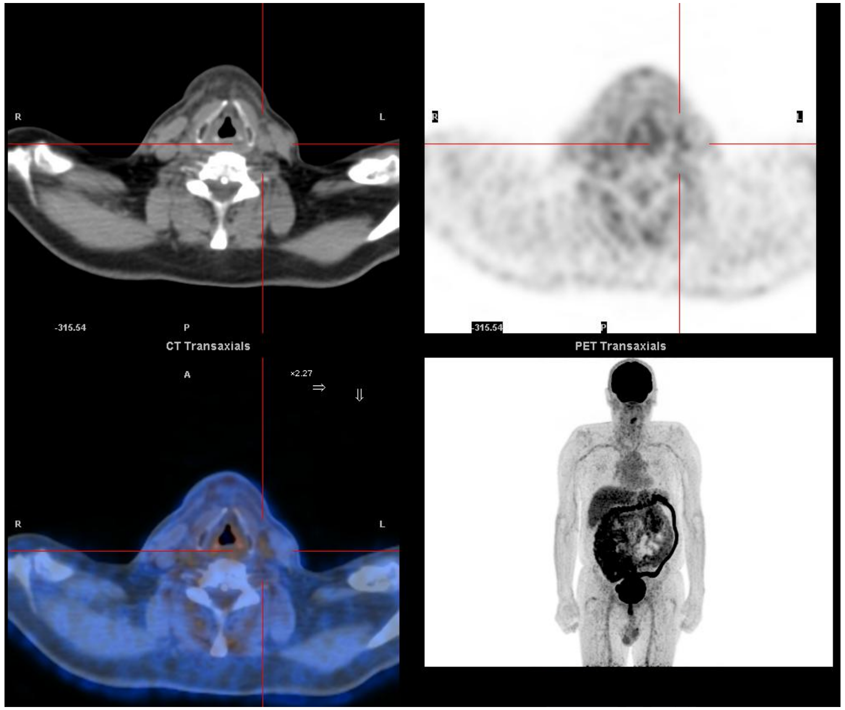

Post-treatment

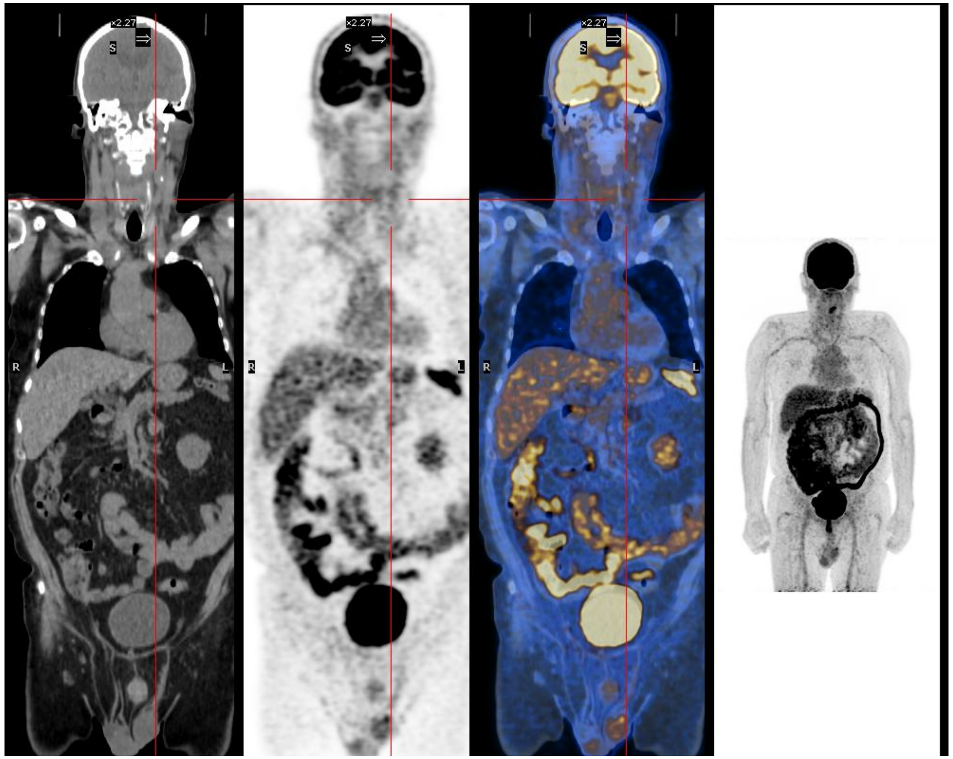

Supplement: Supplementary file 4 — Additional file 4: figure 4. fused FDG-PET/CT images of a 69-year old male with a T4aN2b laryngeal cancer: Nref assessment negative for residual tumor according to all scales [file 41824_2020_77_MOESM4_ESM.pdf]
