# Supplementary material for: Does a 6-point scale approach to post-treatment 18F-FDG PET-CT allow to improve response assessment in head and neck squamous cell carcinoma? A multicenter study
Source: Eur J Hybrid Imaging. 2020 May 26;4:8. doi: 10.1186/s41824-020-00077-9 (PMC8218061; doi:10.1186/s41824-020-00077-9)
Supplement: Supplementary file 5 — Additional file 5: table 1. positive predictive value and corresponding 95% CI of composite TNref for specific scores. PPV: positive predictive value [file 41824_2020_77_MOESM5_ESM.docx]

| **Score** | **PPV estimate** | **95% CI** |
| --- | --- | --- |
| Hopkins score (HS) | 36.8 | 28.1 to 45.5 |
| Cuneo score (CS#1) | 50.0 | 41.0 to 59.0 |
| Cuneo score (CS#2) | 36.8 | 28.1 to 45.5 |
| Cuneo score (CS#3) | 32.0 | 23.6 to 40.4 |
| Deauville score (DS#1) | 36.8 | 28.1 to 45.5 |
| Deauville score (DS#2) | 29.7 | 21.5 to 37.9 |
