# Supplementary material for: Does a 6-point scale approach to post-treatment 18F-FDG PET-CT allow to improve response assessment in head and neck squamous cell carcinoma? A multicenter study
Source: Eur J Hybrid Imaging. 2020 May 26;4:8. doi: 10.1186/s41824-020-00077-9 (PMC8218061; doi:10.1186/s41824-020-00077-9)
Supplement: Supplementary file 6 — Additional file 6: material. FDG-PET/CT analysis [file 41824_2020_77_MOESM6_ESM.docx]

**Supplementary materials and methods**

*FDG PET-CT analysis*

All FDG PET-CT scans had to be performed according to EANM 1.0 procedure guidelines [1], complying with the following criteria: a recommended interval between FDG administration and the start of acquisition within the range of 60 + 10 minutes; plasma glucose level before FDG administration below 140 mg/dL in non-diabetic subjects, below 160 mg/dL in diabetic subjects. All scans had to be acquired through a PET/CT scanner with certified technological qualification. Pending these requirements, the evaluation of included PET-CT images was performed through a centralized digital platform called “Widen” (Web-based image and diagnosis exchange network). The access and use of this programme was guaranteed through an unrestricted grant of AIRO. All patient-specific information was completely anonymized. The following semi-quantitative metabolic parameters were extracted from each scan: Standard uptake value body-weighted (SUV/bw) _max_, where SUV/bw was defined as the concentration of 18F – FDG divided by the injected dose, corrected for the body weight of the patient and radioactive decay at scanning time (SUV = activity concentration/[injected dose/body weight]); SUV/bw _mean_; SUV/bw _peak_, defined as the SUV/bw _mean_ within a 1-cm^3^ sphere centered on the voxels with the highest uptake part of the tumor; Standard uptake value lean-body mass (SUL) where SUL was defined as the concentration of 18F – FDG divided by the injected dose, corrected for the lean-body mass of the patient and radioactive decay at scanning time (SUL = activity concentration/[injected dose/lean-body mass]); SUL/peak, defined as the SUL mean within a 1-cm^3^ sphere centered on the voxels with the highest uptake part of the tumor. All values were reported for the primary tumor (Tref), nodal disease (Nref), liver and mediastinal blood pool (MBP). By definition, with “Tref” we scored the area with highest FDG uptake within the residual primary tumor. With “Nref”, we scored the residual lymph node with highest FDG uptake. The diagnostic accuracy was expressed in terms of: 1) sensitivity, as the proportion of positive cases that are correctly classified by the specific score (true positive rate, TP); 2) specificity, as the proportion of negative cases that are correctly classified by the specific score (true negative rate, TN); 3) positive predictive value (PPV), as the proportion of true positive cases among those with positive result; 4) negative predictive value (NPV), as the proportion of true negative cases among those with negative result; 5) overall accuracy, defined as the proportion of correctly classified cases (TP+TN) among all cases. The difference of SUV/bw parameters between the baseline PETs and final PETs of each patient automatically generated a corresponding delta value. In parallel, an additional evaluation of SUV/bw parameters for treatment response was estimated according to EORTC criteria [2], taking into account that for SUV normalization EORTC recommends to estimate the body surface area. We chose the lesion with the highest 18F-FDG uptake at baseline and measured the same lesion on the follow-up scan. We chose to use SUV_max_ over mean SUV and therefore we did not use isovolumetric measurements of lesion-specific ROIs. Response was classified on each scan according to the 4 categories defined in the criteria. Complete metabolic response (CMR) was complete resolution of 18F-FDG uptake within all lesions, making them indistinguishable from surrounding tissue. Partial metabolic response (PMR) was a reduction in SUV_max_ of at least 25% after more than treatment. Progressive metabolic disease (PMD) was an increase of at least 25% in SUV_max_ or a new 18F-FDG–avid lesion. Stable metabolic disease (SMD) was a response between PMR and PMD.

The computation of percentage variation of SUL values of Trefs and Nrefs allowed us to assess response to treatment also according to PET Response Criteria in Solid Tumors (PERCIST), version 1.0 [3]. As mentioned, PERCIST recommends the use of lean body mass for SUL and the background area was drawn as a 3-cm-diameter spheric ROI in the right lobe of the liver as defined in the criteria. The SUL_peak_ of the hottest lesion on the baseline and follow-up scan was measured. Since the hottest lesions were selected in each scan, the target lesion on follow-up scans was not necessarily the same as the target lesion at baseline. A 1.2-cm-diameter spheric ROI was drawn in the hottest part of the target lesions. The ROIs were placed in the area of the tumor where it resulted in the highest possible mean SUL (SUL_mean_). SUL_mean_ of this ROI was defined as SUL_peak_. The baseline target lesions had to meet the PERCIST 1.0 definition of measurable lesions. The investigators checked that no other lesion could give a higher SUL_peak_. In the follow-up PET/CT scans if SUL_peak_ was decreasing, response was calculated as ΔSUL_peak_ between baseline and actual follow-up divided by baseline SUL_peak_ x 100%. If SUL_peak_ increased, response was calculated as ΔSUL_peak_ between lowest registered and actual follow-up divided by lowest registered SUL_peak_ x 100%. Response was classified on each scan according to the 4 categories defined in the criteria set. CMR was complete resolution of 18F-FDG uptake within all lesions to a level less than or equal to that of mean liver activity and indistinguishable from background blood-pool levels. PMR was a reduction of at least 30% in SUL_peak_ and an absolute drop of 0.8 SUL_peak_ units. PMD was an increase of at least 30% in SUL_peak_ or a new 18F-FDG–avid lesion. SMD was between PMR and PMD.

Finally, to define an objective way to discriminate between moderately and markedly increased FDG uptake in the liver (in particular for to differentiate DS4 from DS5 and HS4 from HS5) we followed the rule reported in Barrington et al [4]. it has been, in fact recommended to apply DS 5 only to uptake at least two times the uptake in normal liver. In this framework, it has been also recommended to confirm visual evaluation by drawing regions in the liver. For this reason a fixed size ROI of 3 cm diameter has been drawn in the right lobe the liver, avoiding the edge and any individual/single ‘hot’ pixels likely to represent noise, to obtain a representative maximum liver SUV.

1. Boellard R, O’Doherty MG, Weber WA, Mottaghy FM, Lonsdale MN, Stroobants SG, et al. FDG PET and PET/CT: EANM procedure guidelines for tumour PET imaging: version 1.0. Eur J Nucl Med Mol Imaging 2010 Jan;37(1):181-200. doi: 10.1007/s00259-009-1297-4.
2. Young H, Baum R, Cremerius U, Herholz K, Hoekstra O, Lammertsma AA, et al. Measurement of clinical and subclinical tumour response using [18F]-fluorodeoxyglucose and positron emission tomography: review and 1999 EORTC recommendations. European Organization for Research and Treatment of Cancer (EORTC) PET Study Group. Eur J Cancer 1999 Dec;35(13):1773-82.
3. O JH, Lodge MA, Wahl R. Practical PERCIST: A Simplified Guide to PET Response Criteria in Solid Tumors 1.0. Radiology. 2016 Aug;280(2):576-84. doi: 10.1148/radiol.2016142043
4. Barrington SF, Mikhaeel NG, Kostakoglu L, Meignan M, Hutchings M, Mueller SP, et al. Role of imaging in the staging and response assessment of lymphoma: consensus of the International Conference on Malignant Lymphomas Imaging Working Group. J Clin Oncol. 2014;32:3048–3058
